# Effects of Banana Plantation Pesticides on the Immune Response of Lepidopteran Larvae and Their Parasitoid Natural Enemies

**DOI:** 10.3390/insects3030616

**Published:** 2012-06-27

**Authors:** Angela M. Smilanich, Lee A. Dyer

**Affiliations:** Department of Biology, University of Nevada, Reno, 1664 N. Virginia St., Reno, NV 89501, USA; E-Mail: nolaclimber@gmail.com

**Keywords:** immune, insect, parasitism, pesticides, banana, caterpillar, melanization, parasitoid, Bayesian, ecology

## Abstract

Basic research on the insect immune response has progressed dramatically within the last two decades, showing that immunity is one of the most effective defenses against foreign invaders. As such, it is important to understand the causes of variation in this response. Here, we investigate the effects of pesticides used in Costa Rican banana plantations on the immune response of the lepidopteran larva, *Caligo memnon* (Brassolinae). In addition, we performed a parasitism survey of the banana plantations and surrounding forests to provide a broader assessment of pesticide effects on parasitoid populations. All caterpillars for the immune assay were collected from two banana plantations and brought to La Selva Biology Station for immune challenge. Individuals were fed leaves from the plantations (pesticide) or leaves from La Selva (pesticide-free), then immune challenged with injected sephadex beads. We found that individuals feeding on pesticide leaves had significantly lower bead melanization compared to individuals feeding on pesticide-free leaves. Nonetheless, the parasitism survey showed that caterpillars from the banana plantations had lower parasitism rates compared to caterpillars from the La Selva forest. This study adds to the growing body of evidence documenting negative effects of pesticides on the insect immune response and on adult parasitoids, and underscores the need for more research at the intersection between ecological entomology and immunology.

## 1. Introduction

From 1954–1973, extensive aerial applications of dieldrin and carbaryl were utilized for 12,000 ha of banana in the Golfito region of southwestern Costa Rica to combat thrips, *Chaetanaphothrips orchidii*. During this time, secondary outbreaks by at least 53 species of caterpillars were frequent in these plantations until the aerial insecticide treatments were halted, and the outbreaking species of Lepidoptera have rarely exceeded economic thresholds since that time [1]. Thrupp [2] hypothesized that the insecticides applied to banana during this time disrupted natural enemies of the lepidopteran defoliators, causing the documented secondary outbreaks. Parasitoids are particularly sensitive to chemical treatments in agriculture [3,4]. Several other studies have examined biological control (hereafter ‘biocontrol’) in banana plantations with a focus on the importance of parasitoids and other enemies, their potential role in biocontrol, and the effects of pesticides on parasitoids [5,6,7]. Here, we focus on the effects of banana pesticides on the immune response of lepidopteran larvae and on parasitoid populations in order to better understand the interaction between pesticides, herbivores, and natural enemies.

One pest attribute that is easily measured and recognized as the best defense against biocontrol agents such as parasitoids and pathogens is the insect immune response [8,9]. This parameter has been underutilized in predictive models and in attempts to increase biocontrol success [10]. Dyer and Gentry [11] developed statistical models based on prey defensive characters to predict success of existing biocontrol programs, but this study did not include the immune response as a predictor variable because it has been measured infrequently for common outbreaking species in natural and applied ecosystems. In a recent review by Stanley [10], the case is made for utilizing the vast increase in our knowledge of the insect immune response as a means for making better biocontrol programs. For example, many studies have shown that dietary chemistry can have a dramatic effect on the performance of the immune response [12,13]. By understanding the causes of variation in the insect immune response, it may be possible to create biocontrol programs that are environmentally neutral. In this paper, we examine potential effects of synthesized chemicals (pesticides) on the immune response of caterpillars feeding in banana plantations, as well as the effects of these pesticides on the parasitoid populations. 

Parasitoids are the most important source of mortality for many species of herbivorous insects [14], and thus have the potential to act as strong selective agents on the evolution of their hosts’ defenses. Caterpillars have a variety of defenses against parasitoids (reviewed by [15]). Pre-oviposition defenses include colorations and morphologies that prevent parasitoids from finding or attacking their hosts; and behaviors, such as thrashing, regurgitating, or dropping off the plant [8,16,17,18,19], that may repel or confuse parasitoids.The primary post-oviposition defense against parasitoids is the immune response, specifically, encapsulation. Encapsulation is a cellular response in which hemocytes congregate around eggs or larvae of parasitoids in order to asphyxiate them [20,21]. Encapsulation is usually followed by a melanization response where melanin is deposited on the hemocytes and in the process of synthesizing melanin free radicals such as quinones are produced, which are cytotoxic to the parasitoid egg or larva [20]. 

The immune response is a complex and resource heavy process [22,23], and there are many causes of variation including both genetic [24,25,26] and environmental sources [13,27,28]. As previously mentioned, plant chemistry is a source of variation and can have a strong effect on the strength of the immune response. In general, pesticides may negatively affect the immune response as caterpillars will be naïve to detoxifying these synthetically produced compounds. Thus, we hypothesize that caterpillars feeding on pesticide-exposed leaves will have a reduced immune response compared to those feeding on pesticide-free leaves. If the immune response is compromised by pesticides, then it is possible that parasitism rates will be higher in banana plantations where pesticides are used than in nearby forests without pesticides. However, negative effects of pesticides on parasitoid physiology may override any gain in parasitoid success due to a suppressed immune response. Insecticides are frequently more toxic to parasitoids than to host species [29,30,31,32,33,34,35,36]. The mechanism behind this result is unclear; however, it is suggested that the endocrine disrupting action of pesticides is a likely candidate. Many of the products applied to high input banana plantations are considered to be endocrine disruptors [37], particularly the fungicides and insecticides. For example, a number of studies have identified chlorpyrifos and carbofuran as potent endocrine disruptors [37,38,39]. Consequently, our second hypothesis is that parasitism rates will be lower in banana plantations compared to nearby forests. To test these hypotheses, we collected caterpillars from two banana plantations in Costa Rica and reared them on leaves with pesticides and pesticide-free leaves. We also collected and reared caterpillars from the plantations to obtain parasitism rates.

## 2. Materials and Methods

### 2.1. Study System

**Figure 1:**
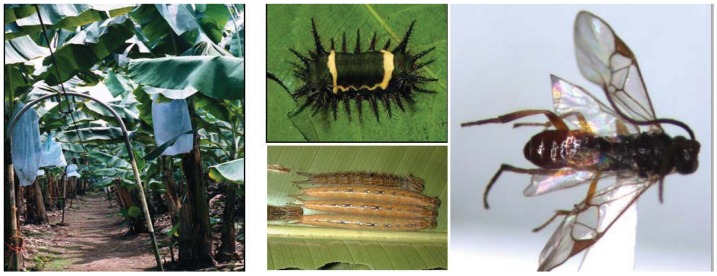
A subsample of our model study organisms. Clockwise from left: banana plants with fruit bags that contain chlorpyrifos; *Acharia nesea* (Limacodidae) larva; *Cotesia *sp. (Braconidae) adult parasitoid; *Caligo memnon* (Nymphalidae) larvae.

Bananas (*Musa* spp.,Musaceae, Figure 1) are the largest export crop in Costa Rica and are the most popular fruit in the United States (reviewed by [7]). In 2009, banana imports exceeded 14 million tons in the USA-Canada, European Union, and Japan; and Costa Rica is the third top exporter of bananas world-wide behind Ecuador and Philippines [40].Commercial banana cultivation practices currently depend upon regular applications of a variety of fungicides, herbicides, insecticides and nematicides to maintain productivity (see [5,6,7] for complete list of pesticides used). We focused our caterpillar collection on two farms near Puerto Viejo, Sarapiqui, Costa Rica. Rebusca is a conventional input farm, while Penjamo is a moderate-input farm (Table 1).

**Table 1:** Locations used for caterpillar collection. The main measurable difference between the sites were that conventional plantations used at least 5 applications per year of 6–8 kg ha^−1^ Terbufos (a nematicide), the moderate plantations used 1 application per year of 4–5 kg ha^−1^ Terbufos, and no pesticide inputs were applied at La Selva (See [5,6,7] for complete list of pesticides used).

Location	Region (Cantón)	Area (Ha)	Pesticide Input
Penjamo	Sarapiquí	148	Moderate
Rebusca	Sarapiquí	102	Conventional
La Selva	Sarapiquí	1,600	None

### 2.2. Immune Challenge

We investigated how exposure to agro-chemicals (herbicides, fungicides, nematicides, and insecticides) affected the encapsulation and melanization response of *Caligo memnon* (Nymphalidae, Brassolinae) caterpillars. These caterpillars are commonly found in banana plantations and feed on plants in the genus *Musa *(Musaceae) and *Heliconia* (Heliconiaceae). During June and July 2004, *C. memnon* caterpillars were collected from both Rebusca and Penjamo and brought back to La Selva Biology Station for immune challenge. For each sampling trip, half of the collected individuals were changed to feed on pesticide-free leaves from the forest at La Selva. To challenge the immune response, sephadex beads, 40–120 µm, (Sigma-Aldrich) were injected into the hemoceol of 3rd–5th instar caterpillars [9,41]. Before injected, the beads were dyed with Congo Red. Ten beads in Ringers solution were injected into the hemolymph of each caterpillar using a glass Pasteur pipette fashioned into an injection syringe [13]. The injection site was covered with Second Skin^®^ adhesive to repair any damage. The caterpillars were allowed to continue feeding on their respective diets for 24 hr after injection and were freeze-killed. Beads were recovered by dissection and photographed using a camera mounted on a dissection microscope focused at 80×. Since the beads were dyed red before injecting them into the caterpillars, we quantified melanization by measuring the red value (r-value, Adobe Photoshop ver. 6.0), a scale ranging from 0–255, where 0 = pure gray, and 255 = pure red, for each bead. The mean r-value for all the beads from each caterpillar was statistically compared between treatments. The r-value was transformed into percent melanization (1-(r-value/maximum r-value)) where the maximum r-value is 255 for a non-injected, unmelanized bead. 

### 2.3. Statistics

A Bayesian approach was used to analyze these data using informative priors from previous experiments with *Caligo memnon *[9]. Bayesian analysis produces statistics that estimate the probability of the hypothesis given the data [P(hypothesis)|data] and incorporates prior parameter estimates to create posterior probability distributions. The mean and variance of melanization from prior encapsulation experiments with *C. memnon* were used as informative priors for the caterpillars fed La Selva leaves (mean = 74, variance = 474). For the caterpillars fed pesticide exposed leaves, we utilized an effect size from immune response experiments that examined responses to leaves treated with secondary metabolites *versus *non-treated leaves [13] to calculate the pesticide prior (mean = 61, variance = 474). To estimate model parameters, we used Markov chain Monte Carlo (MCMC) simulation where each step in the chain estimates melanization based upon the prior data. We used a 2000 step burn-in period followed by the 10,000 step MCMC to generate the posterior density distribution for the melanization of pesticide and pesticide-free individuals. We calculated posterior probabilities of the null hypothesis (no difference in encapsulation) by quantifying the frequency that the difference was equal to or greater than zero in each step of the post burn-in MCMC. For example, if pesticide-free (F) individuals have a higher melanization than the pesticide (P) individuals (F-P > 0) across 99% of the post-burn-in MCMC steps, we can conclude that the probability that melanization in pesticide-free caterpillars is equal to melanization in pesticide caterpillars is 0.01 [42,43]. Bayesian analyses were performed in SAS v.9.3 using PROC GENMOD.

### 2.4. Parasitism Survey

From 2004 to 2006, we collected and reared caterpillars from banana plantations to obtain parasitism rates. The method used by our laboratory in the past [7,6,44] for collecting caterpillars in agriculture were utilized. Plantation managers were given a brief fact sheet about the project, and asked to fill out a very simple sheet with information about management practices at their farm. Caterpillars were collected in plastic bags and taken back to La Selva for rearing. Caterpillars were housed in an ambient lab and checked daily for parasitoids or adults. Fresh leaves from the banana plantations were added to the bags as needed. Once parasitoids emerged, they were stored in the freezer until they could be pinned. Parasitism frequency per species was calculated as the total number of parasitized individuals divided by the total number of results: (total # parasitized)/((total #adults + total #parasitized)).

## 3. Results and Discussion

### 3.1. Immune Challenge

**Figure 2:**
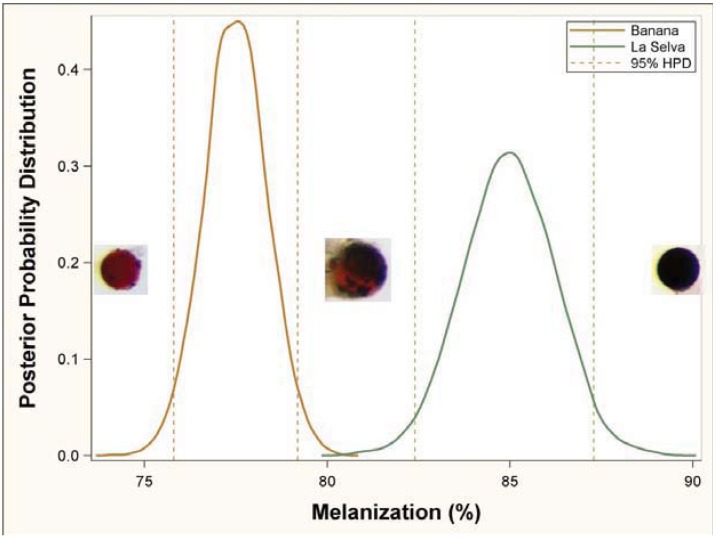
Posterior probability distribution of the melanization response for caterpillars feeding on banana leaves (pesticide) and La Selva leaves (pesticide-free). Dotted vertical lines reference the 95% highest probability density (HPD) interval. Non-overlapping HPD intervals for the two distributions indicate that the mean melanization for each population is different. Sample bead images across the distributions are representative of low, medium, and high levels of melanization.

MCMC simulations for the melanization response produced non-overlapping posterior probability distributions between the pesticide (banana) caterpillars (95% HPD (highest probability density: 1.94–2.61) and the pesticide-free (La Selva) caterpillars (95% HPD: 4.66–11.4) (Figure 2), indicating a difference in the melanization response (MCMC for the null, P < 0.0001). The 95% HPD interval or credibility interval is similar to 95% confidence intervals, but in this case indicates that there is a 95% chance that the population mean actually occurs within the calculated interval. Here, we found that the 95% HPD intervals for the banana and La Selva populations do not overlap, indicating a support for our hypothesis and a significant difference in the mean melanization response between groups. 

**Figure 3:**
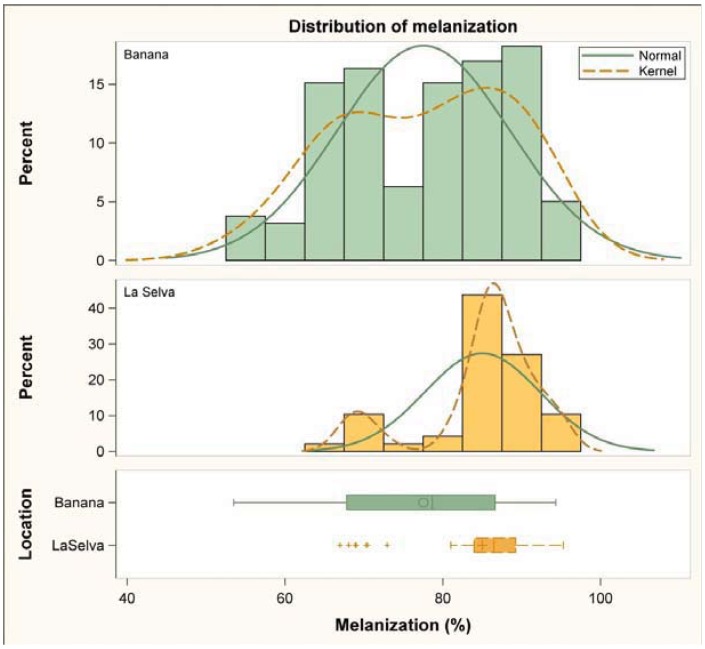
Histogram of the melanization response for the individuals feeding on pesticide leaves from the banana plantations (top panel) and those feeding on pesticide-free leaves from the La Selva forest (middle panel). Box plot of melanization from the two locations is on the bottom panel.

**Figure 4:**
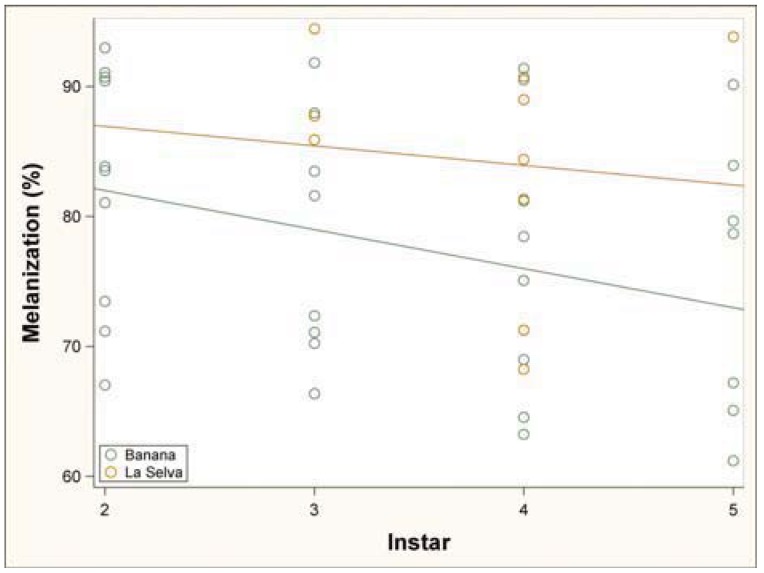
The percent melanization across four instars. Early instars had a slightly higher melanization score than later instars.

Caterpillars feeding on the pesticide-free leaves (mean = 85.0, SD = 7.2, n = 48) had a higher melanization response compared to the caterpillars feeding on the pesticide leaves (mean = 77.5, SD = 10.9, n = 159) (Figure 3). When we examined the change in melanization over instars, we found that early instars had a slightly higher melanization score compared to later instars (2^nd^ instar mean = 82.5, SD = 9.2; 3^rd^ instar mean = 81.2, SD = 9.6; 4^th^ instar mean = 77.9, SD = 9.8; 5^th^ instar mean = 77.4, SD = 11.9) (Figure 4).

### 3.2. Parasitism Survey

Parasitism rates were highest at La Selva, the pesticide-free location, followed by Penjamo and Rebusca (Table 2), the moderate and conventional pesticide plantations. At each location, parasitism was dominated by tachinid flies. This was especially true at both plantation sites, where tachinids accounted for 78% (Penjamo) and 81% (Rebusca) of total parasitism (Table 2). The species with the highest parasitism rate was the saddleback caterpillar, *Acharia nesea* (Limacodidae) (Table 3). These results corroborate earlier studies that predict highest levels of parasitism by tachinids, followed by braconids then other parasitic hymenoptera [6,11]. Models by Dyer and Gentry [11] also predict highest levels of parasitism by tachinids for generalists, such as *Acharia* and lowest levels of parasitism by tachinids for specialists, such as *Caligo*.

**Table 2:** Number of caterpillars collected at each site as well as the number of adults and parasitoids reared. Not all caterpillars collected resulted in adults or parasitism. Highest parasitism occurred at La Selva, a forested site with no pesticide application, followed by Penjamo, a moderate-input site, and lastly Rebusca, a conventional-input site.Parasitism frequency per species was calculated as the total number of parasitized individuals divided by the total number of results: (total # parasitized)/((total #adults + total #parasitized)).

Location	Caterpillars Collected	Adult	Parasitism	Parasitoid ID	Parasitism by Taxa
**La Selva**	542	276	26%	Tachinidae	60%
				Braconidae	24%
				Ichneumonidae	4%
				Chalcididae	4%
				Eulophidae	2%
				Hymenoptera	3%
**Penjamo**	932	606	18%	Tachinidae	78%
				Hymenoptera	22%
				Braconidae	7%
**Rebusca**	1031	708	14%	Tachinidae	81%
				Hymenoptera	8%
				Braconidae	4%
				Ichneumonidae	1%

**Table 3:** The most abundant caterpillar species collected from all sites along with the number of each species collected, the number reared to adult, and the percent that were parasitized. Not all caterpillars collected resulted in adults or parasitism.

Species	Caterpillars Collected	Adult	Parasitism
***Caligo memnon* (Brassolinae)**	1,211	808	12.2%
***Antichloris viridis* (Erebidae)**	863	564	20.8%
***Opsiphanes tamarindi* (Brassolinae)**	285	148	27.0%
***Acharia nesea* (Limacodidae)**	118	19	65%
***Acharia hyperoche* (Limacodidae)**	50	35	10.2%

### 3.3. Discussion

Results from the immune assay support our hypothesis that pesticides have a detrimental effect on the immune response of caterpillars. In this case, our inference is limited to the encapsulation and melanziation in *Caligo memnon* from two specific ecosystems in Costa Rica. Nevertheless, these results may be relevant to other insect species in similar ecosystems, as the evidence for the immunosuppressant activity of pesticides accumulates [45]. Since injections were performed within 24 h of *C. memnon* collection, pesticide residues on leaves most likely affected the inducible components of the immune response. That is, since individuals fed pesticide-free leaves after injection were initially collected from the banana plantations, it appears that the negative immune effects are reversible in a short period of time, with the immune suppression persisting on individuals that continued feeding on leaves with pesticide. 

The most common pesticides found on banana plantation leaves are fungicides and a nematicide [5]. The fungicide is aerially deposited on leaves, thus herbivores are exposed both through the cuticle and during feeding. The nematicide is granular and applied at the base of the banana stem, where it seeps into the soil and is absorbed by the roots. The nematicide then becomes systemic in the plant and herbivores are again exposed during feeding (Dyer, unpublished data). Since treatment individuals were feeding on leaves collected from plantations, they were ingesting not just one pesticide, but likely two or more. This gives rise to the possibility that there could be a synergistic effect of the banana pesticides on the immune response. Recent studies show that synergistic effects of secondary metabolites may be a common mode of action by which plants deter herbivores [46,47,48]. While we did not explicitly test for synergy in pesticides, the possibility cannot be ruled out that there may be a combinatorial effect of pesticides on the insect immune response.

The effects of banana plantation leaves on the caterpillar immune response suggest that parasitic Hymenoptera might be more successful when their hosts are feeding on plantation leaves. However, in plantations not only will herbivores be exposed to pesticides, but also parasitic flies and wasps by the aerial spray and through nectar feeding. Results from the current study and in Dyer 2005 [6] demonstrated that parasitism by Hymenoptera increases as levels of pesticide decrease (natural forest > moderate input > conventional input; Table 2), and the increase is accompanied by a concurrent increase in proportion of parasitism by tachinid flies. In addition, the decrease in abundance of parasitoids at the banana plantations compared to forest sites indicates that although caterpillars are immune compromised, it is not benefiting parasitoid populations (as measured by percent parasitism). The first possible mechanism to explain these patterns is a potential increase in host availability for tachinids because of lower numbers of hymenopterans. Empirical [49] and theoretical [50] studies as well as reviews [51] have documented the importance of interspecific competition between parasitoids utilized for biological control. The second possible explanation, which could act in concert with the first, is that tachinids are released from control by hyperparasitoids. Several species of *Brachymeria *(Chalcididae), an important parasitoid genus in our banana plantations [6], are facultative hyperparasitoids of tachinids [52,53,54]. Although *Brachymeria *have not been reported to parasitize the Tachinidae observed in the study, they have been reared from other Lepidoptera parasitized by *Lespesia aletiae*, the numerically dominant tachinid observed in this study [55,56]. However, our collection and rearing techniques did not allow us to determine whether any of the parasitic Hymenoptera reared were hyperparasitoids. Finally, Matlock and de la Cruz (2002) [5] found apparent negative effects of chlorpyrifos, organophosphates, and carbamate nematicides on hymenopteran parasitoid communities in banana plantations. Our results clearly corroborate this negative association between high pesticide use and hymenopteran parasitism rates of banana caterpillars, but both the effects on encapsulation and the positive association between tachinid parasitism and pesticide use demands further study.

## 4. Conclusions

The artificial division between basic and applied entomology has been discussed at great length [57,58,59], and it is clear that recognizing the similarities between natural and managed systems will facilitate the improved management of both. Results from this study show that pesticides can suppress the immune response of *C. memnon* and decrease natural enemy populations, much in the same way that plant secondary metabolites suppress the immune response of herbivores in natural systems [9,13]. Any attempts to manage for continued suppression of Lepidoptera outbreaks in Costa Rican banana should take into account the natural associations between such caterpillar defenses and the level of parasitism by different insect taxa. Our study allowed us limited insight into how agricultural chemicals may modify host-enemy interactions. In a recent review, James and Xu 2012 [45] lament the lack of empirical studies addressing the effects of pesticides on insect immunity. Future research should expand the investigation to how specific herbicides, nematicides, fungicides, or insecticides impact all parameters of the insect immune response as well as all stages of parasitic flies and wasps in banana and other agricultural ecosystems.
